# A program theory for a continuing professional development program to enhance primary care nurses’ mental health assessment practices

**DOI:** 10.3389/frhs.2026.1887809

**Published:** 2026-08-26

**Authors:** Ariane Girard, Marie-Hélène Lemée, Sarah Lafontaine, Jean-Daniel Carrier, Marie-France Langlois, Hélène Clabault, Luz Piedad Arroyave, Johanie Gagné

**Affiliations:** 1 School of Nursing, Faculty of Medicine and Health Sciences, Université de Sherbrooke, Sherbrooke, Québec, Canada; 2 Departement of Psychiatry, Faculty of Medicine and Health Sciences, Université de Sherbrooke, Sherbrooke, Québec, Canada; 3 Department of Medicine and Continuing Education Center, Université de Sherbrooke, Sherbrooke, Québec, Canada; 4 Nursing Directorate, CIUSSS Centre-Ouest-de-l’Ile-de-Montréal, Montréal, Québec, Canada; 5 General Services Directorate, CIUSSS Estrie-CHUS, Sherbrooke, Québec, Canada

**Keywords:** clinical assessment, clinical supervision, continuing professional development, mental health, nursing, primary care, reflective practice

## Abstract

**Aim:**

To develop and test the relevance of a program theory underpinning a continuing professional development (CPD) program designed to support primary care nurses in adopting evidence-based mental health assessment practices.

**Background:**

Primary care nurses play a key role in identifying and responding to mental health needs, yet often report limited confidence and support in conducting assessments. A CPD program grounded in reflective practice and clinical supervision was co-created to address these gaps. Program design included a program theory representing the selected training processes and their expected outcomes.

**Design:**

Theory-driven evaluation using relevancy testing with a mixed-methods approach.

**Methods:**

Data collection included questionnaires based on the Ability, Motivation and Opportunity (AMO) framework at baseline, post-training, and 4–6 months follow-up; semi-structured interviews; observational field notes; and a logbook. Research team members analyzed qualitative data thematically and quantitative data descriptively.

**Results:**

Fourteen primary care nurses from two healthcare centres in Quebec participated (*n* = 13 completed). The program enhanced nurses’ ability (knowledge, skills), motivation (confidence and professional engagement), and perceived opportunities at the individual level. Participants reported integrating new practices, including systematic screening, goal-setting, and follow-up, indicating behavioural change. Results suggest reflective and clinical supervision mechanisms that may have explained these outcomes and that enrich the initial CPD program theory.

**Conclusions:**

A CPD program integrating reflective practice and clinical supervision can strengthen conditions for evidence-based mental health assessment in primary care. Sustained practice change, however, requires alignment with organizational support processes.

## 1 Introduction

Half of Canadians will experience a mental illness by the age of 40 (1), with common mental disorders such as anxiety and depression being the most prevalent (2). Common mental disorders are the second reason for long-term disability worldwide (2) and affected people have a variety of needs relating to their safety, disease management, lifestyle, and recovery (3, 4). At the first line of mental health services, primary care nurses (PCNs) play an important role in assessing people with mental health problems and identifying those at risk of common mental disorders (5–7). PCNs should provide comprehensive mental health assessment and adopt a recovery-oriented perspective contributing to patients’ empowerment and self-management (8–11). While mental health assessment is covered by initial nursing training (12), PCNs often report feeling uncomfortable assessing their patients’ mental health needs and insufficiently supported to do so (6, 13–15). Contributing factors include limited knowledge and skills specific to primary care (14, 15), unclear role expectations (6, 13), and insufficient access to tailored continuing professional development (CPD) (14, 15). Therefore, mental health assessment requires specific attention in the CPD of PCNs.

Quality CPD requires learners to be able to reflect on their practice and build on their acquired knowledge with new evidence-based information (16, 17). Moreover, accessing clinical supervision from experienced colleagues is particularly important for integrating evidence-based mental health nursing practices in primary care settings (5). However, little is known concerning the optimal structure and expected impact of a continuing professional development program based on reflexive practice and clinical supervision. Responding to needs emerging from real-world practice settings, we collaboratively developed such a training program and proposed an initial theoretical model underlying its expected effects (18). We hypothesized that the program's short-term outcomes would involve increasing PCNs’ ability (knowledge and skills), motivation (desire to accomplish a task), and opportunity to provide quality mental health assessment in their work environment according to best practices, conditions often referred to as the AMO (Ability, Motivation, and Opportunity) Framework (19). The AMO framework is commonly used in human resource management to understand the factors that shape worker performance (19). In this paper, we report on the validation of the initial theoretical model (thereafter: program theory).

A program theory, or a theory of change, is essential for understanding how a program or an intervention may produce its results (20). A program theory consists of conceptualizing the relationships between a program and its outcomes. It allows for clarification of the “black box” of an intervention and for going beyond outcomes (20). Assessing outcomes alone is not enough to capture the complexity of a program and its context (21). As many contextual factors may influence the outcomes of an educational or CPD program, it's important to clarify what works in which circumstances (22). Evaluation of a program theory allows to understand the causal mechanisms that may contribute to outcomes. In the context of professional education and CPD, demonstrating outcomes is particularly challenging as programs or interventions are often complex, i.e., involve many actors and components interacting with the context. The validation of a CPD program theory enables the tackling of the complexity of demonstrating educational outcomes.

The aim of this study was to develop and test the relevance of the program theory for a CPD program aimed at enhancing the adoption of evidence-based mental health assessment practices. The specific objectives were to: (1) explore and analyze the perceived outcomes of the CPD program; and (2) suggest potential mechanisms that may have contributed to these outcomes. In the context of this study, the term “mechanism” is conceptualized as underlying processes and participant reactions to the program activities (23).

## 2 Context of the study

The study was conducted in Quebec (Canada), where PCNs primarily practice in family medicine groups. Family medicine groups follow an organizational model in which family physicians collaborate with interdisciplinary teams—including nurses, social workers, and psychologists —to address patients’ primary healthcare needs (24). Within this model, PCNs commonly manage patients with chronic conditions such as diabetes, cardiovascular and pulmonary diseases, and cognitive disorders (25, 26). This project originated from a request by two PCN clinical advisors, who sought the research team's help to co-develop a training program to enhance PCNs’ mental health assessment practices.

## 3 Initial program theory

**Figure 1:**
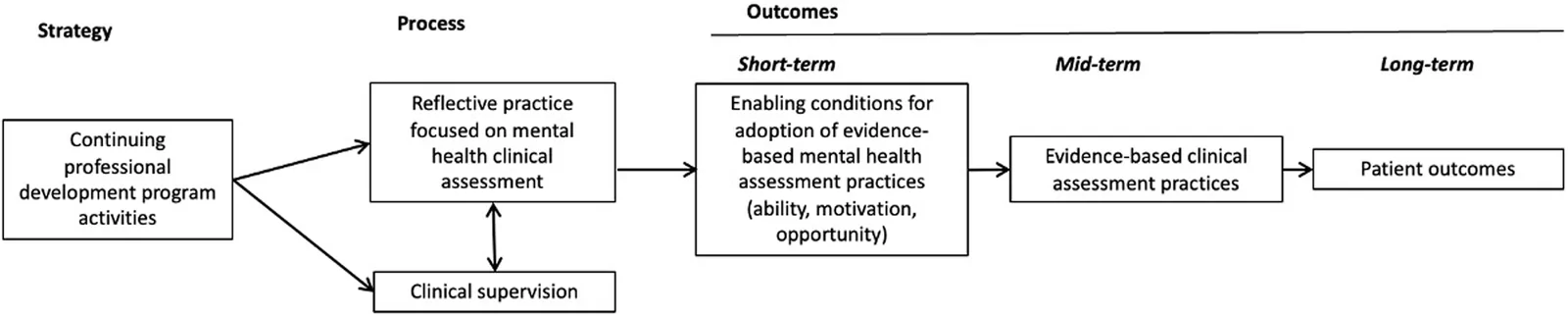
Initial continuing professional development program theory (18).

A program theory was developed with stakeholders and end users to articulate the relationships between CPD program activities (i.e., the training strategy), underlying processes, and expected outcomes (18). Using the AMO framework as a useful lens to understand how professional behaviours are shaped in the workplace (19), we hypothesized the CPD program activities, through the process of reflective practice oriented toward clinical assessment, to contribute to strengthening the conditions (ability, motivation, opportunity) conducive to adopting evidence-based mental health assessment practices. These changes were expected to lead to improvements in clinical practice (mid-term outcomes) and, ultimately, patient outcomes (long-term outcomes). Additionally, based on prior literature on clinical supervision, we conceptualized clinical support as a structured, formal process that supports reflective practice and professional development (27), thereby constituting a second process that reinforces the reflective practice process. We conceptualized clinical supervision and decision-making in complex situations. The initial program theory model is presented and detailed in the published protocol (18) and summarized in Figure 1.

## 4 Characteristics of the CPD program

### 4.1 CPD program objectives

The CPD program aimed to: (1) explain the importance of clinical mental health assessment, (2) identify additional opportunities to assess mental health needs, (3) conducing mental health assessments and screening, (4) identify relevant resources to support one's mental health assessments, and (5) critically analyze one's clinical practices to identify areas for improvement (18).

### 4.2 Characteristics and roles of the facilitators

Four categories of facilitators were involved: clinical supervisors, nursing coordinators, mental health nurses experts, and research team members. Clinical supervisors (*n* = 4) were expected to attend all group activities and remain attentive to PCNs’ needs and challenges in implementing evidence-based mental health assessment practices. One objective of the main project was to clarify their role to further develop training and materials adapted to their needs. Nursing coordinators (*n* = 2) oversaw program implementation within their respective settings and were responsible for scheduling activities, recruiting PCNs, selecting and supporting clinical supervisors, participating in group activities, identifying implementation challenges, communicating issues to management, and contributing to knowledge transfer. Mental health nurse experts (*n* = 4) facilitated two group case discussions and provided *ad hoc* support to PCNs and clinical supervisors. The research team led program development, facilitated the research process, designed educational materials, and supported facilitators in their roles.

### 4.3 Structure and educational tools

The six-week CPD program, included both individual and group activities, ranging from 20 min to two hours per week (Table 1). The program was structured around three core components of reflective practice: (1) self-assessment, (2) action, and (3) follow-up. Group sessions were delivered via Microsoft Teams© in groups of three to eight participants. In week 1, PCNs reviewed a training guide (4) and summary and completed a reflective questionnaire comparing their practice with evidence-based practices to identify areas for improvement. In week 2, participants engaged in an online group discussion of three fictional clinical cases facilitated by a clinical supervisor to support integration of learning. In weeks 3 and 4, participants selected a challenging clinical case from practice and completed a structured case report to prepare for a co-development activity. In week 5, a co-development session facilitated by a mental health nurse expert used a structured guide (28) and followed four steps: (1) presentation of clinical cases and identified challenges; (2) group selection of two cases for in-depth discussion; (3) guided case analysis using probing questions; and (4) collective brainstorming of solutions (28, 29). In week 6, participants completed reflective questions on their learning journey and barriers encountered in implementing mental health assessment practices, including role clarity, access to mental health specialists, and availability of validated screening tools. A final group discussion enabled sharing of reflections and the identification of practice improvement opportunities, facilitated by a nursing coordinator and clinical supervisor.

**Table 1:** Summary of the training program structure and activities.

Reflective goal	Period and activity type	Activity description	Duration	Materials
Self-assessment	Week 1 Individual activity	- Reading a mental health training guide on mental health assessment practices - Reflecting on one's own practice through guided questions, including identification of improvement goals	- 90 min	- Training guide (30 pages including annex) - Summary of the training guide (2 pages) - Reflective questionnaire on LimeSurvey (3 questions)
	Week 2 Online group activity	- Discussing fictional clinical cases based on the training guide	- 90 min	- Animation guide - PowerPoint presentation including three fictive cases
Action	Week 3–4 Individual activity	- Identifying a challenging real-life clinical case and completing a summary sheet for co-development	- 20 min	- Case report form (word.doc)
	Week 5 Online group activity	- Participating in a professional co-development group	- 180 min	- Animation guide including a description of each step
Follow-up	Week 6a Individual activity	- Answering reflective questions on integrating evidence-based mental health assessment practices into one's clinical setting	- 30 min	- Reflective questionnaire on LimeSurvey (4 questions)
	Week 6b Online group activity	- Discussing challenges related to the adoption of evidence-based mental health assessment practices	- 90 min	- Animation guide

Educational materials were developed by the research team and reviewed by co-researchers and additional collaborators with expertise in many fields such as pedagogy, mental health nursing, and professional development. A patient partner contributed to the revision of materials to ensure relevance and alignment with patient perspectives. The program was accredited by the “ Centre de formation continue de la Faculté de médecine et des sciences de la santé de l’Université de Sherbrooke ” (the CPD office of the first author's faculty). Learners received 6.5 accredited hours upon completion.

## 5 Methods

### 5.1 Program design

A living lab approach was used to co-develop the training program with end users (PCNs), nursing advisors, and managers from two regional healthcare centres. This approach engages end users and stakeholders as research partners in the co-creation of innovations tailored to their needs and practice contexts (30). The living lab approach includes five stages: (1) planning, (2) conception, (3) prototyping, (4) final development, and (5) deployment. Each stage involves specific activities such as co-creation, exploration, experimentation, and evaluation (30). This paper reports findings from the project's prototyping phase, which focused on testing the relevance of the program theory while experimenting the program activities with end users. An initial version of the program theory and implementation model (activities and resources) was developed during the conception phase.

### 5.2 Research design

A theory-driven evaluation using the relevancy testing approach (21) was conducted to develop and test the CPD program theory. The relevancy testing approach consists of testing the program theory with stakeholders at an early stage of program design. This approach determines whether the program addresses the intended problems and examines outcomes as experienced by participants (21). It supports program refinement by assessing the relevance of targeted outcomes, identifying potential mechanisms, and examining acceptability from participants’ perspectives (21).

A mixed-methods design with qualitative emphasis was employed (31). Mixed-methods approaches are commonly used in implementation research because they enable a comprehensive understanding of intervention processes and outcomes (32). Qualitative data provide insight into contextual factors influencing outcomes and acceptability, whereas quantitative data are used to measure outcomes and examine hypothesized changes (32). In this study, quantitative data were used to assess perceived outcomes (Objective 1), to describe the study context, and to triangulate findings with qualitative data. The Standards for Reporting Qualitative Research (SRQR) guided the presentation of the methods section (33). Findings related to program acceptability are reported separately (Girard et al., submitted).

### 5.3 Participants recruitment

PCNs were recruited for participation by their clinical advisor or manager, who distributed a summary of the program. To be eligible, participants had to (1) work in a family medicine group and (2) be available and willing to engage in the program for its full duration. A purposive maximum variation sampling strategy was used (34). Specifically, recruitment sought to include PCNs with diverse profiles in terms of practice context, mental health assessment practices, and professional characteristics (e.g., self-efficacy regarding mental health competencies) (18). Potentially interested nurses were referred to the research coordinator (MHL). A research assistant provided detailed study information, obtained informed consent, and supported enrollment. Participants then received access to an introductory meeting, online training materials, and group activities via Microsoft Teams©.

### 5.4 Data collection

Data were collected at four time points: before, during, immediately after, and four to six months after the program (program experimentation: November 2023; data collection completion: June 2024). Qualitative data collection was guided by the Ability, Motivation, and Opportunity (AMO) framework, which is commonly used to assess innovation adoption in healthcare (19). Quantitative data are presented to support qualitative findings.

#### 5.4.1 Qualitative data

Qualitative data collection tools are described in detail in the published research protocol (18). Collected data included:

- Observational reports. Research team members (SL, AG, MHL, and HC) and nursing coordinators observed three group activities without participating. Detailed field notes documented engagement, interactions, reactions to content, and emerging learning processes, contextualizing participants’ experiences and informing iterative refinement of the interview guide. Observational findings were triangulated with interview data and used to support the identification of potential mechanisms during the final stage of analysis (Objective 2).
- Logbook. The research team maintained a logbook to document decisions, challenges, reflections, and observations throughout data collection and analysis, supporting reflexivity, team communication, and iterative improvements.
- Semi-structured interviews. Individual interviews (45–60 min) were conducted by the researcher coordinator (MHL) immediately post-program and at four to six months. The interview guide explored participants’ experiences of program activities and perceived outcomes, using open-ended questions with prompts to facilitate in-depth exploration. Specifically, the guide for the first interview comprised two sections: (1) conditions supporting the adoption of evidence-based mental health assessment practice and perceived program effects, and (2) program acceptability and feasibility. The first section was informed by the AMO framework (19) and previous work on the role of PCNs in caring for individuals with mental health concerns (6). The second section was informed by the Kirkpatrick training evaluation model (35), particularly its learning and reaction dimensions. The guide for the six-month post-interview was informed by the AMO framework. It explored practice changes since PCN's participation and the challenges they may face in maintaining the adoption of evidence-based mental health assessment practices. Both interview guides were revised by one nurse coordinator. Interviews were conducted via Microsoft Teams©, recorded, and stored on a secure institutional server (SharePoint©).

#### 5.4.2 Quantitative data

Online LimeSurvey© questionnaires, inspired by the AMO framework (19), were completed at baseline (T0), immediately post-training (T1), and at four to six months follow-up (T2) (18):

- *Sociodemographic data*: Eight multiple-choice and short-answer question items (e.g., gender, age, years of practice since graduation, family medicine group experience, work status, experience with mental health patients).
- *Continuing education:* Two dichotomous/multiple-choice questions on mental health training in the past year.
- *Organizational data:* Two dichotomous/multiple-choice questions assessing clinic size and affiliation.
- *AMO outcomes:* Ability (8 items), motivation (2 items), and opportunity (6 items), rated on a four-level Likert-style scale (“completely disagree” to “totally agree”). For instance, opportunity items included: “My work environment facilitates my ability to provide high-quality clinical assessments to individuals with mental health issues” and “Clinical assessment of individuals with mental health issues is clearly part of my role and responsibilities”. AMO items were developed for this study. Validated instruments were not used at this stage because no consensus exists regarding the most appropriate tools. Findings were intended to inform the selection of relevant AMO-related outcomes and guide future use of validated instruments.
- *Frequency of mental health assessment:* Five-level Likert scale question, ranging from “never” to “many times per day”: “In the last three months, how often have you conducted a clinical nursing assessment with a person experiencing a mental health issue (related to a diagnosis of mental disorder or not)?”

T1 and T2 questionnaires repeated *AMO* and assessment frequency measures.

### 5.5 Data analysis

Qualitative data were transcribed, de-identified, and analyzed using Nvivo14© with thematic analysis following Braun & Clarke's (36) six steps: familiarization, coding, theme development, review, definition/naming of themes, and analysis writing. All interviews were conducted in French. For the purpose of this manuscript, verbatim excerpts were translated into English by research professionals using Google Translate and DeepL, followed by review for accuracy. Initial coding was informed by AMO framework (19). Analysis combined deductive and inductive approaches to allow the emergence of unanticipated findings. Two research professionals conducted the analysis. Three interviews were independently coded by both analysts, who met after each coding round to discuss discrepancies and refine coding decisions. A codebook was developed to ensure consistent interpretation of codes and themes. Analysts met regularly throughout the process to discuss emerging codes, methodological questions, and analytic decisions, which were documented in an audit trail. Any unresolved discrepancies were discussed with the principal researchers. Quantitative data were analyzed using descriptive statistics (frequencies and proportions) in SPSS© (IBM, 2023). When item-level responses were missing, we calculated the percentages from the available data. We calculated the results of participants who completed the survey at least twice (before and after the program). Two datasets were created: one including participants who completed questionnaires at T0 and T1, and a second including participants who completed questionnaires at T0, T1, and T2.

Data integration followed a convergent approach, guided by the Universal Design for Learning (UDL) framework (37–39) to support the integration of findings and better understand how program activities may have influenced outcomes. Originally developed to guide the design of inclusive learning environments (40), the UDL framework adopts a neurobiological perspective and identifies three types of learning networks: affective, recognition, and strategic (38). The affective networks related to the “why of learning,” including the environment's emotional and motivational significance. The recognition networks correspond to the “what of learning” and the transformation of information into knowledge. The strategic networks reflect the “how of learning” and support planning, organizing, and initiating actions (38).

### 5.6 Ethical considerations

Ethical approval was obtained from the Ethics review board of the *Centre Intégré Universitaire de Santé et de Services Sociaux de l'Estrie-CHUS* (MP-31-2024-5109). Funders had no role in study design, data collection, analysis, manuscript preparation, or journal selection.

## 6 Results

Results are presented into three sections: (1) contextual data, (2) training program outcomes (Objective 1), and (3) mechanisms underlying program activities contributing to these outcomes (Objective 2).

### 6.1 Contextual data

#### 6.1.1 PCN learners’ sociodemographic characteristics and level of participation

A total of 14 PCNs were recruited (Table 2). Program completion rate was 93% (*n* = 13/14), with one learner dropping out after Week 2. Among completers, only one missed one session (Week 5) due to illness. Participants’ clinical mental health experience ranged from less than six months to more than five years, with most reporting two to five years. Of the 14 learners, 36% (*n* = 5/14) had received mental health training in the past two years (e.g., suicide risk assessment: 21%, *n* = 3/14). Participation in interviews (*N* = 20) was high immediately post-program (93%, *n* = 13/14) and moderate at follow-up (50%, *n* = 7/14; four to six months post-program).

**Table 2:** Sociodemographic and organizational characteristics of PCN learners.

Characteristics	Number (total = 14)
Individual characteristics	
Age (years)	
• 18–29	1
• 30–39	6
• 40–49	6
• 50–59	1
Gender	
• Women	11
• Men	3
Higher level of education	
• Bachelor's degree	12
• Master's degree	2
Years of experience as a PCN	
• <4	3
• 4–6	5
• 7–10	3
• 11–15	1
• ≥16	2
Organizational characteristics	
Clinic affiliated with a university	
• Yes	8
• No	6
Number of patients registered at the clinic	
• <10,000	6
• 10,000–19,999	5
• >20,000	3

#### 6.1.2 PCN learners’ clinical mental health assessment practices

PCNs reported encountering patients with mental health needs across various clinical contexts (e.g., chronic disease follow-up, cognitive assessment, vaccination, ADHD follow-up). Although consultations were often unrelated to mental health, PCNs identified symptoms such as anxiety, social isolation, suicidal ideation, depressive symptoms, motivational difficulties, and functional impairments. More than half of PCNs (54%, *n* = 7/13) reported alternating mental health follow-ups with family physicians. Practices varied between clinical settings depending on organizational structures and role definitions. In some settings, nurses were involved when psychotropic medications were initiated or adjusted; in others, care organization remained unclear, particularly regarding role sharing and interdisciplinary collaboration.

### 6.2 CPD program outcomes (objective 1)

Results are based on an integrated analysis of quantitative and qualitative data. As only 8 of 14 PCNs completed all three questionnaires (T0-T1-T2), quantitative findings focus on this subgroup. We report short-term outcomes (ability, motivation, opportunity), followed by mid-term outcomes (adoption of mental health assessment activities) and long-term outcomes (perceived patient impacts).

#### 6.2.1 Ability, motivation, and opportunity (short-term outcomes)

##### 6.2.1.1 Ability

The training supported the development and consolidation of competencies, including structuring assessments and documentation, using mental health terminology, understanding the scope of practice, recognizing patient needs, applying recovery-oriented approaches, using assessment tools, and engaging in reflective practice (Table 3). Ability was described as both knowledge and an increased awareness in practice: “*I think that because I'm more conscious of it, I'm starting to make my notes a little clearer and more consciously as well.”* (ID4). Some participants reported refinement of pre-existing competencies: “*I think it was already there, but perhaps I explored it more deeply. I may be more comfortable exploring the mental health aspect.” (ID10*). PCNs who completed all questionnaires indicated a small variation in their perception of having the knowledge required to provide a high-quality clinical assessment for people with mental health issues [75% (*n* = 6/8) at least agreeing at T0, 87, 5% (*n* = 7/8) at T1, and 100% (*n* = 8/8) at T2]. Similarly with qualitative data, we observe a positive variation in their perception of being comfortable to establishing a nursing care plan focused on the needs of a person with mental health issues immediately after the program [71% (5/7) disagree at T0, and only 14% (1/7) at T1]. However, this perception changed negatively at 6 months post-program [57% (4/7) disagree at T2].

**Table 3:** PCNs’ ability (knowledge and skills) – short-term outcomes.

Theme	Participant quotes
Structuring mental health assessments and clinical documentation	*"To know what I'm going to talk about. Already to be more aware of the content of an assessment, the questionnaire and follow-up, the tools that are available too […]”* (ID14)
	*"It [the training guide:* http://hdl.handle.net/11143/21451*] allows me to structure my interview and sometimes, when […] it goes off on a tangent, I don't forget important elements. […] I think that the fact that I am more structured, the fact that I am able to identify things more quickly, has helped me, it helps me caring for patients.” (ID13)*
Using mental health-specific terminology	*"You know the training guide; the vocabulary is there. It's easier for me to transcribe my idea with the right vocabulary”* (ID13)
Understanding the scope of nursing practice in mental health care	*"It made me aware of the importance of mental health, and the importance that a nurse clinician plays in the assessment, management and follow-up of patients with mental health issues.”* (ID6)
	*"That's when I saw how much more in-depth the other nurses in other family medicine groups were in their mental health follow-ups [it] made me think, “Wow, okay, we're in a different place here.” Is there really more we can do than just say, “are you okay?”* (ID14).
Recognizing patients’ mental health needs	*"Awareness of the importance of mental health, even if I think I was already aware of it, but (…) to be sensitive to detecting it and screening for it, then to prioritize afterwards, […] being aware that it's omnipresent practically all the time.”* (ID12)
Applying a recovery-oriented approach	*"Everything related to recovery wasn't something I was familiar with. So for me, that was something new. “(ID13)*
Identifying and using relevant tools and resources (e.g., screening tools)	*"I feel more comfortable because I know the tools, the symptoms, and the questions to ask.”* (ID5)
Engaging in reflexive practice	*"You know, before, I might have had some reflection, but it was less structured, so now by having some sort of structure, a reference document for me it's easier to do some self-reflection*.” (ID13)
	*"I think more about the way I did my evaluation*.” (ID4)

##### 6.2.1.2 Motivation

Motivation was reflected in participants’ increased confidence, their intention to improve their competencies and clinical services, and their desire to share their newfound knowledge (Table 4). PCNs who completed all questionnaires showed consistently high intrinsic motivation to intervene for people with mental health issues, going from 87.5% (*n* = 7/8) at T0 to 100% (*n* = 8/8) at T1 and T2. Because baseline motivation was already high, opportunities for measurable improvement were limited (ceiling effect). Qualitative findings nevertheless suggest that the program may have reinforced participants’ motivation to engage in ongoing professional development.

**Table 4:** PCNs’ motivation – short-term outcomes.

Theme	Participant quotes
Intention to improve personal competencies and clinical services	*"One of my resolutions for January would be to create a specific mental health note that I can refer to, you know, certain phrases, certain templates if you will, and then draw inspiration from the guidance manual, the summary there, and import tools that are not in my note*.” (ID7)
	*"It made me a ‘hungry’ [for mental health]. I heard the other day that there was a training course that seemed pretty good with the Quebec Order of Nurses, on anxiety disorders, so I'll check it out soon. I'd say I'm good for my training hours this year, but next year, it might be on my bucket list.”* (ID9)
	*"Me and ID9, we also decided to meet in a few weeks concerning the training. We want to say, ‘OK, where are we going, what have we done, what have we changed,’ all that stuff, to come back to it. Because, you know, we're in it now, so we're doing well, and we're working on it. But I don't want it to fall by the wayside*.” (ID13)
Desire to share knowledge with peers and patients	*"We spread the word among our colleagues so that they would be aware of what we can do to help them, not just me, but my other colleagues as well, in terms of mental health*.” (ID12)
	*"I have two colleagues here who have less experience in family medicine groups, and it happened that they presented me with situations where I suspected there was something wrong with the person's mental health. Just talking about it with my colleagues and grappling with abstract concepts related to mental health assessment, I find that this leads to a reflective approach.”* (ID11).
Increase sense of competence and confidence	*"I feel better equipped, more competent. Because I have more tools, I'm not stuck with a hot potato after all. I feel competent to hold the space for patients who are experiencing these issues*.” (ID7)
	*"[…] feel more competent, and then when you go to give your summary to the doctor, you feel like you've really done a thorough assessment, like you've fulfilled your role as a nurse.” (ID12)*

##### 6.2.1.3 Opportunity

Findings related to opportunity were more nuanced. Participants reported increases in their perceptions of professional support, teamwork, trust, and access to clinical supervision by peers (Table 5). However, organizational barriers persisted, particularly instability and lack of clarity in referral processes: “*The most frustrating thing […] is when we really have to make a mental health referral […] it changes all the time, and we never know if we’re sending it to the right place.*” (ID4). Nevertheless, participants generally agreed that their work environment facilitates high-quality mental health assessment, and this perception didn't change with time for PCNs who completed all questionnaires [(T0: 87.5% (*n* = 7/8); T1: 75% (*n* = 6/8); T2: 87.5% (*n* = 7/8)].

**Table 5:** PCNs’ opportunity – short-term outcomes.

Theme	Participant quotes
Perceived support for improving practice	*"I liked that she [the Nursing Coordinator] was there because, ultimately, after the project, she will still be our point of reference in the field. […] And the fact that she was able to hear all of this […] the issues, the questions, knowing how we are going to translate that into action in the field. […]”* (ID13)
	*"I think ID29, who is a* community nurse advisor*, took notes so that she could possibly offer us training in the future that could be adapted or useful for our mental health practice.”* (ID6)
Improved teamwork and trust among colleagues	*"I think I do that more. I use them [social workers] more as a resource if I ever have questions about a case. I send questions to social workers more often than I did before.” (ID5)*
	*"I try to be more proactive when I suggest a plan to the doctor to follow up with a phone call after a certain amount of time, to show the added value of what I can do. By showing that I understand what needs to be monitored?” (ID11)*
	*"I know that I am more able to guide my intervention with these patients and also to document it. And if I send these well-written, well-documented reports to several doctors every time a situation arises, I wouldn't be surprised if they sent the patients back to me and said, ‘Look, I see what you're capable of. Now, can you do [alternating] follow-ups.'”* (ID14)
	*"I now know that I can contact other professionals. I feel more supported in terms of follow-up*.” (ID2)
Provision of peer support	*"Seeing that group process, I thought, ‘Well, with my colleagues, how can I not be intimidating with all of this?’ ‘how can I make people feel comfortable?’ […] When I have a colleague, who is going to tell me about a clinical situation, I try to reformulate […] ‘so you did this intervention’ ‘Great, you know’ ‘Ah well done’.”* (ID11)
	*"I am more and more comfortable with mental health cases, and even my colleagues come to see me to discuss mental health cases too, because we still see them often*.” (ID5)

#### 6.2.2 Adoption of mental health assessment activities (mid-term outcomes)

Follow-up interviews (four to six months post-program) identified changes in practice, including: (1) screening for individuals at risk of mental disorders, (2) goal setting with patients, and (3) follow-up and monitoring of mental health conditions (Table 6). While most PCNs reported conducting mental health assessments *almost weekly* at both T0 and T2, the proportion conducting assessments *almost daily* varied from 25% (*n* = 2/8) at T0 to 62.5% (*n* = 5/8) at T2. However, one PCN who had *never* conducted assessments at baseline did not show improvement post-program and did not complete follow-up questionnaires.

**Table 6:** Adoption of mental health assessment activities – mid-term outcomes.

Theme	Participant quotes
Screening individuals at risk of mental disorders	*"I definitely do a little more screening. If someone comes in with high blood pressure, sometimes it turns into something related to mental health.* […] *I hesitate less, I ask the question. Then you know, I have the PHQ 2 that we had explored as a clinical tool that comes to mind quickly*.” (ID9)
Setting goals with patients and developing action plans	*"It is easier for me to establish, let's say, goals with my patient. By considering the objectives, the recovery perspective, I found it easier to get involved and to offer a goal to the patient, [to find a goal] with them. To find with a more precise objective.”* (ID13)
	"*We can bring a little more nursing aspect to all the other aspects of mental health […], you don't need to be medicalized or, you know…* *in the non-pharmacological, without being a psychoeducator, without being a nutritionist, without being a kinesiologist either, I think we're able to give people guidelines, to set goals. And then, to bring that into something quite concrete as well*.” (ID9)
	"*I sort of assessed, ‘Do you have a plan?’, ‘Do you have the resources to accomplish that kind of plan'?”* (ID5)
Conducting follow-up and monitoring mental health conditions	"*I feel more comfortable with follow-ups, like how often I should see them. I think that, in general, I am more involved with mental health patients now*.” (ID2)
	"*I do follow-ups [about their mental health] when I see the patient again for their chronic illness*.” (ID10)
	*"I find that I [assess] more completely. I don't stay as shallow. I assess patient's safety quite a bit more when I collect data. In a way,* *…* *I think I was assessing it [mental health], but less systematically.”* (ID12)

#### 6.2.3 Perceived patient outcomes (long-term outcomes)

Participants perceived that patients receiving care from trained nurses experienced greater continuity of care: “*It's about ensuring good continuity between team members. Not just […] with me from one meeting to the next. Yeah, it allows us to more effectively detect whether there's a deterioration or an improvement. So yeah, it's continuity […]”* (ID 11). Some participants also perceived changes in their communication style and believed that patients may have experienced greater satisfaction with care, including feeling listened to and understood: “*I find the way I talk to patients has changed too, like having more of an empathetic voice […]. Then 2 patients also told me after triage: ‘Ah, I felt really good with you’. Within 5 min, they opened up a lot because they said they felt good. There's even one patient who told me, ‘You're really good’, ‘Thank you!'”.* (ID5). Participants further suggested that these changes may have strengthened nurse-patient partnerships and supported patient empowerment:

“I think it's about knowing how to manage them (the patient) better and making them feel taken care of, too. I think it's an added value for everyone to assess the patient first. [..] I think that in the end, the patient is better served, that we work better as a team there, and then when we leave, we know better.. that we speak the same language, too.” (ID13)

“Perhaps we weren't asking the questions as well, let's say […] In such a situation in the past, how did you cope? name what helped him so that he himself could find the solutions, also through empowerment.” (ID12)

### 6.3 Reflective practice and clinical supervision mechanisms that may have contributed to outcomes (objective 2)

Based on data analysis, we suggest mechanisms through which program activities may have contributed to outcomes, particularly short-term outcomes (ability, motivation, opportunity). CPD activities, especially the training guide and case discussions with experts and peers, may have fostered reflective practice by activating affective, recognition, and strategic learning networks (37). These mechanisms supported the adoption of evidence-based mental health assessment practices. Six clinical supervision mechanisms emerged from data analysis: (a) representation of evidence, (b) support for self-reflection, (c) emotional support from peers, (d) problem-solving through case discussions, (e) knowledge sharing with peers and experts, and (f) planning for organizational change and improvement. Based on findings, we hypothesize that these clinical supervision mechanisms may have facilitated reflective practice and activated reflective mechanisms related to learning networks of the Universal Design for Learning framework. The Universal Design for Learning framework provided a useful lens to organize reflective mechanisms according to their primary learning functions (Table 7). We use the definition of learning networks to categorize reflective mechanisms identified by participants. The following sections describe how specific program activities and clinical supervision mechanisms may have contributed to reflective mechanisms within each learning network. Supplementary Material S1 summarizes the reflective practice and clinical supervision mechanisms associated with each program activity and their potential contribution to short-term outcomes.

**Table 7:** Suggested reflective mechanisms.

Affective networks	Recognition networks	Strategic networks
- Developing self-awareness through reflection on one's practice - Preparing for group activities - Normalizing one's experience through comparison with peers - Taking ownership of roles and responsibilities - Experiencing emotions (positive or negative) that support learning	- Structuring assessment practices - Validating knowledge and clinical practice - Developing mental health-specific vocabulary - Acquiring new knowledge - Comparing practices and knowledge with peers - Connecting evidence with experiential knowledge - Consolidating knowledge	- Developing templates for data collection and clinical documentation - Identifying opportunities for mental healh assessment - Analyzing one's practice and identifying areas for improvement - Identifying solutions to improve nursing practice and care organization

#### 6.3.1 Affective networks

Most activities contributed to sustained engagement and motivation: “*The further we got, the more engaged I became, and it was so interesting that I learned more and more as we went along.”* (ID2). The combination of individual preparation (e.g., reading, self-reflection) and group activities supported engagement: “*the fact of having things to prepare. […] with the pre- and post-activities […] we knew where we were going, we had to reflect.” (ID8).*

Group activities (week 2 and 5) enabled sharing of experiences and knowledge with peers, normalization of one's situation, taking ownership of one's role and responsibilities, and emotional expression. For example, comparing herself to her peers during this first group meeting had the effect of demotivating one learner “[…] *‘Oh my God, I felt like, for all the time I've been practicing, I felt incompetent. [..] So that's it, I'd say that after the first meeting, I was like – Ho boy, I'm not sure I'm going to do it.” (ID10).* At the same time, peer comparison also helped normalize difficulties*: “You feel less alone […] You see that others are facing certain challenges or […] experiencing situations […] with patients […] that can be more difficult or […] confrontational.” (ID3*).

#### 6.3.2 Recognition networks

The training guide and case discussions may have activated the recognition networks (week 2 and 5). The structured progression from general to specific content facilitated learning: “*I think it worked well to really go back over the documents, I think it was a good sequence because it allowed us to familiarize ourselves with the document first, then discuss it, and then move from the general to the more individualized.” (ID2)*. Program activities supported consolidation of knowledge: “*I think it [knowledge] has become more consolidated. […] there are things that we know but don't necessarily use, and this has kind of dusted them off and brought them to the forefront […].” (ID11)*. Increased knowledge contributed to building confidence: “*[…] with the training [..] It expands your knowledge in general. So when you have more knowledge, I think at the same time, you have more confidence in yourself to assess.”* (ID5).

The training guide may have supported both acquisition and validation of knowledge:

“The guide we were given at the beginning, already, I learned a lot, like on the assessment, because it's really complete [..] I learned that there are sites and programs you can use to manage your symptoms yourself[..]. I have even more tools to share with my clientele.” (ID2)

“[…] it [knowledge] was pretty well acquired, but to see it and then read it in its entirety, it was good.” (ID3)

Fictional case discussions may have contributed to connections between experience and evidence: “*I really enjoyed that meeting, particularly because we were able to make connections with the guide.” (ID6)*. The co-development group meeting seems to contribute to the acquisition, validation, and consolidation of knowledge by sharing experiences and solving problems with mental health experts. One learner said that the mental health nurse practitioner “*was able to provide guidance on things that we don't necessarily know about.”* (ID3). One learner explained that she learned “[…] *from the experience of others by proxy to approach certain cases, just from the tips we gave each other during co-development” (ID9)*. The richness of the discussions was enhanced by the fact that the learners had different areas of expertise: “*I think that the co-development meetings were really the key to success, because that's where there was the most exchange between novices and.. let's call them ‘experts’. So yeah, I think it can be really interesting to see, ‘Oh, OK, we can do that. We can go that far.'” (ID11)*.

#### 6.3.3 Strategic networks

Almost every activity may have contributed to activating the strategic networks, as program activities supported goal setting, planning for change with peers, and practice improvement. Activities may have contributed to participants’ motivation and perceived opportunity to adopt evidence-based mental health practices. For example, some PCNs developed their own documentation templates after reading the training guide and attending the co-development meeting:

“I loved the way it was structured [the guide]. Okay, so that really helped me understand how to build a note. […] it gave me ideas so I wouldn't forget anything, resources too, lots of resources. [.]” (ID7)

“Based on that meeting (co-development) and the knowledge I had gained, that's when I created my own note templates […] I also added all the questionnaires we could use, along with their interpretations, to our software.” (ID14)

Problem-solving with peers through case discussions (week 2 and 5) enhanced identification of assessment opportunities: “*seeing lots of assessment opportunities, […] makes me think and say, ‘Oh, maybe in that situation, I could have seen that’. Then, after that, you think about it […] during your next appointments, and it becomes more natural.” (ID8*). Preparation for co-development also fostered critical reflection: “*The process to get to the co-development activity allowed me to take a critical look at my general practice, to realize that I wasn't consistent with the GAD7 and PHQ9.” (ID11)*.

*Finally,* group activities contribute to identifying ideas for improving nursing practices*: “Seeing what others are doing, then thinking about what I could do better, that's what I find most motivating. Because if we think we're doing everything perfectly, it's not going to motivate us; […] Seeing what changes we can make gives us a little boost […]” (ID5).* By comparing oneself to other learners during co-development meeting, PCNs realized what their organization did well or not: “*we can sometimes put into perspective the differences from one environment to another depending on the resources that are available or the interests or lack thereof of the doctors, or precisely what is offered in terms of services as a clinical nurse in a family medicine group, it can vary greatly.” (ID9)*.

Planning for change and discussing challenges with peers and clinical supervisor also contribute to fostering opportunity “*[…] I felt a sense of sharing and a desire to evolve, change, and help the practice. So for me, that's really encouraging. You know, when you're able to take something from that and turn it into something else. […] we could all put our ideas together and ultimately do something with them. Not just, you know, write things down on paper or whatever.” (ID13)*.

## 7 Discussion

**Figure 2:**
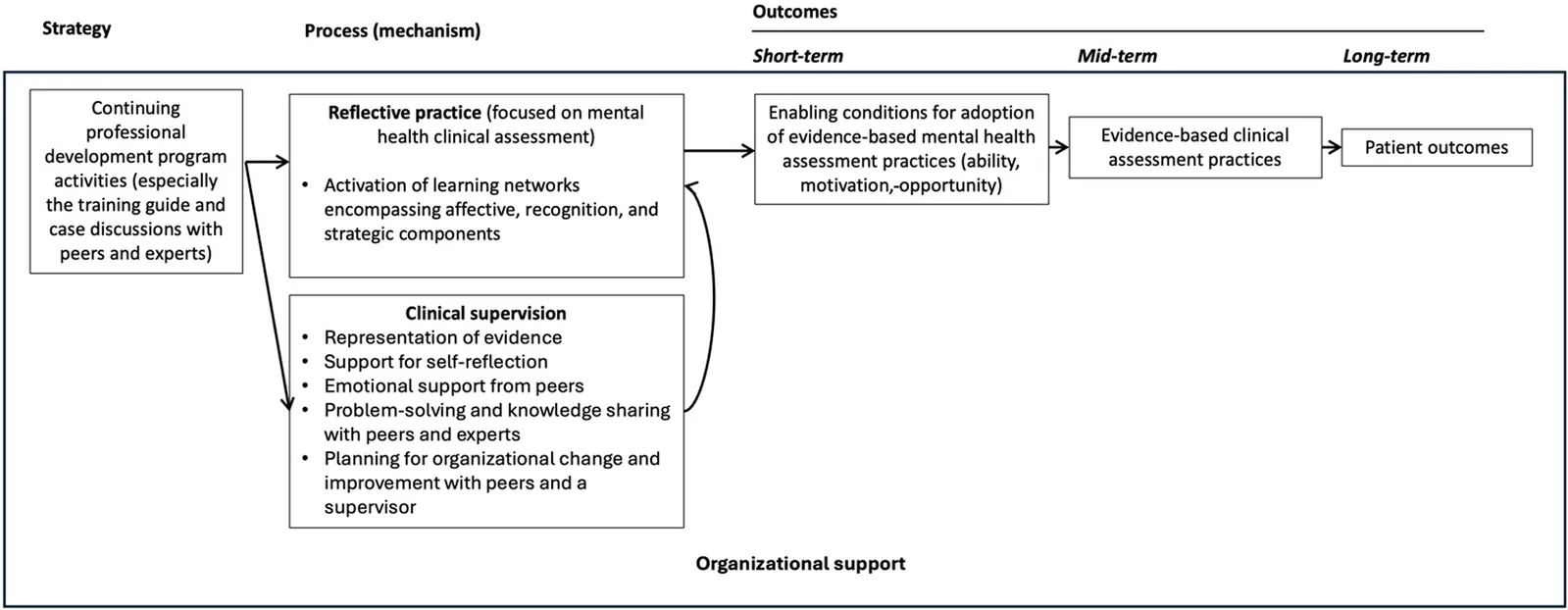
Revised continuing professional development program theory.

To our knowledge, this is the first study to examine the program theory of a continuing professional development program aimed at improving the adoption of evidence-based mental health assessment practices. Findings suggest that program activities enhance the ability, motivation, and opportunity to adopt best practices at the individual level, and that learners’ experiences are consistent with the short-term outcomes resulting from the activation of affective, recognition, and strategic learning networks. However, results related to opportunity at the organizational level were more limited. Moreover, findings suggest that the initial program theory was relevant but lacked some important insights about the possible mechanisms underlying the program's enhancement of ability, motivation, and opportunity at both individual and organizational levels. Thus, we propose a revised CPD program encompassing reflexive and clinical supervision mechanisms (Figure 2). These mechanisms help clarify how program activities may have contributed to outcomes. This is particularly important for the dissemination of such a program and its ongoing improvement, as mechanisms may serve as levers to optimize outcomes and the implementation process.

Organizational support was not a process targeted by our CPD program. However, contextual factors—conceptualized as opportunity—play a critical role in shaping organizational behaviours and the implementation of evidence-based practices (41). In our study, ability and motivation often interacted to foster opportunity at the individual level. For instance, participants improved peer support and perceptions of teamwork following the program. However, program activities did not appear to enhance organizational-level opportunity practices, such as job design. This was particularly evident among PCNs who were not previously involved in mental health follow-up and for whom the role and responsibilities lacked clarity. To sustain the adoption of evidence-based mental health assessment practices, the program should incorporate activities that explicitly target organizational support, such as engaging decision-makers and providing ongoing reinforcement or technical support. Organizational support processes should be developed in collaboration with key decision-makers, including nursing leaders, human resources directors, and clinical managers. Consistent with findings from a systematic review of continuing professional development, CPD should be conceptualized not merely as a learning activity but as an organizational change intervention that uses education as a mechanism (42).

Our results suggest that activities that allow for reflective practice, such as reading a training guide and discussing cases with peers and experts, may activate reflective mechanisms consistent with the learning networks of the Universal Design Learning framework (37). Our results also align with some items of the reflective practice questionnaire, such as feeling more confident, the desire to improve, and the capacity to apply a reflexive practice after the program (43). The potential relationships between the three learning networks (affective, recognition, and strategic) and reflective practice can help clarify how reflective practice is operationalized. Additionally, reflective supervision enhances reflective practice (44). In our study, participants mentioned six clinical supervision mechanisms supporting reflective practice: (a) representation of evidence, (b) support for self-reflection, (c) emotional support from peers, (d) problem-solving through case discussions, (e) knowledge sharing with peers and experts, and (f) planning for organizational change and improvement with peers and clinical supervisors. Those mechanisms are consistent with previous knowledge and guidelines on clinical supervision (27, 45), as well as with the literature on facilitation of evidence-based practices (46, 47). For instance, being involved in organizational change and quality improvement initiatives and receiving emotional support are key components of the EQUIP model, a nursing supervision model developed by the National Health Service in England (45). Summarizing evidence or providing access to evidence-based knowledge, promoting experience and knowledge sharing, and promoting critical reflection are facilitation strategies commonly used to enhance the adoption of evidence-based practices (46, 47). Further study should investigate the causal relationship between reflective and these supervision or facilitation mechanisms and outcomes.

Ability (e.g., self-confidence, competencies) and motivation are commonly targeted outcomes of continuing professional development (42, 48). In this study, these outcomes were achieved; although PCNs’ motivation was already high at baseline, the training helped sustain this level. Ability and motivation are recognized as mediators between human resource management practices (e.g., clinical supervision) and employee performance (19). Even though participants reported some changes in their practices, assessing mid- and long-term outcomes, including patient outcomes, remains challenging due to the lack of systems for systematically collecting data on nursing practices and patient health. Further research is needed to address these measurement challenges (42). Moreover, given the complexity of healthcare systems that was reflected in participants’ input, we believe that the program theory should incorporate a systems approach, defined as “[…] *a way of addressing health delivery challenges that recognizes the multiplicity of elements interacting to impact an outcome of interest and implements processes or tools in a holistic way.*” (49).

### 7.1 Strengths and limitations

This study reports findings from the prototyping phase of a living lab research project. The co-construction of the training program with PCNs and stakeholders increases its relevance to primary care context (30). The qualitative analysis provided in-depth insights into PCNs’ experiences and supported the interpretation of quantitative findings. Methodological rigor was strengthened through triangulation of data sources and methods, consultation with experts, and iterative analysis within the research team (50). Interview guides were iteratively refined, and peer debriefings contributed to analytic credibility. A reflexive journal was maintained to document assumptions and potential biases, and methods were described in detail.

Limitations included the small sample size (*n* = 14) and limited participant heterogeneity, which may affect transferability. Participants were predominantly women aged 30–49 years, with a bachelor's degree or higher and 0–10 years of primary care experience. Although recruitment was not stratified, this is consistent with exploratory research and feasibility considerations (51). Only eight participants completed all questionnaires (T0-T1-T2), and these individuals were highly motivated and often had prior mental health experience. Additionally, we didn't use validated tools to measure AMO outcomes. Quantitative findings were therefore interpreted alongside qualitative data and should not be considered in isolation. Finally, desirability bias may have influenced interview responses, as some participants were familiar with members of the research team.

All interviews were conducted by the same interviewer, except for one, which was conducted by the first author (AG) because the research coordinator (MHL) personally knew the participant. Using a single interviewer may have strengthened consistency across interviews; however, it may have also introduced biases related to the interviewer's background, assumptions, and communication style. Several strategies were implemented to mitigate these risks, including the use of a structured interview guide that was iteratively refined with the research team, regular debriefing meetings with the first author following interviews, and the completion of reflexive notes after each interview to document observations, thoughts, and potential sources of bias.

## 8 Conclusion

This study provides one of the first empirical examinations of a CPD program theory aimed at enhancing primary care nurses’ adoption of evidence-based mental health assessment practices through reflective practice and clinical supervision. The six-week program may have activated reflective mechanisms—affective, recognition, and strategic networks—consistent with the Universal Design for Learning framework. These mechanisms contributed to improved ability, sustained motivation, and enhanced perceptions of opportunity at the individual level. Reported impacts included increased confidence, more structured assessments, greater reflexivity, and improved collaboration, with some effects persisting four to six months post-program. However, changes in organizational-level opportunity were limited. PCNs working in contexts with unclear roles or care pathways experienced fewer practice changes, underscoring the importance of organizational conditions in sustaining evidence-based practices. These findings suggest that while reflective practice and clinical supervision are essential components of continuing education, they are insufficient in isolation to drive sustained practice change in the presence of structural barriers. Future iterations of the program should incorporate explicit organizational support processes to strengthen opportunity and sustainability. Further research is needed to refine these processes and evaluate their impact on practice and long-term patient outcomes.

## Data Availability

The datasets presented in this article are not readily available because they contain personal information. Requests to access the datasets should be directed to ariane.girard2@usherbrooke.ca.
