# Supplementary material for: A program theory for a continuing professional development program to enhance primary care nurses’ mental health assessment practices
Source: Front Health Serv. 2026 Aug 26;6:1887809. doi: 10.3389/frhs.2026.1887809 (PMC13553543; doi:10.3389/frhs.2026.1887809)
Supplement: Supplementary file 1 [file Table1.docx]

| **ACTIVITIES OF THE TRAINING PROGRAM** | **CLINICAL SUPPORT MECHANISMS** | **REFLECTIVE MECHANISMS** | **OUTCOMES (AMO)** |
| --- | --- | --- | --- |
| **Week 1**   - Reading a mental health training guide on assessment practices - Answering reflective questions, including the identification of improvement goals | - Representation of evidence - Support of self-reflection | **Affective network**   - Developing self-awareness through reflection on one’s practice - Prepare for group activities | **Motivation**  **Ability**  **Opportunity** |
|  |  | **Recognition networks**   - Structuring assessment practices - Validating one’s knowledge and practice - Developing mental health-specific vocabulary - Acquiring knowledge - Comparing one’s practice and knowledge |  |
|  |  | **Strategic networks**   - Developing a template for structuring data collection and clinical documentation |  |
| **Week 2**   - Discussing fictional clinical cases based on the training guide | - Emotional support from peers - Problem-solving through case discussions - Sharing knowledge and experiences with peers and experts | **Affective network**   - Normalizing one’s experience through comparison with others - Taking ownership of one’s role and responsibilities - Experiencing emotions (positive or negative) conducive to learning | **Motivation**  **Ability**  **Opportunity** |
|  |  | **Recognition networks**   - Acquiring knowledge - Comparing one’s practice and knowledge - Making connections between evidence and experiential knowledge |  |
|  |  | **Strategic networks**   - Identifying solutions to improve nursing practice or care organization |  |
| **Week 3-4**   - Identifying a complex clinical case and completing a summary sheet for co-development | - Support for self-reflection | **Affective networks**   - Prepare for group activities | **Motivation**  **Opportunity** |
|  |  | **Strategic networks**   - Analyzing one's practice and identifying areas for improvement |  |
| **Week 5**   - Participating in a co-development meeting | - Emotional support from peers - Problem-solving through case discussions - Sharing knowledge and experiences with peers and experts | **Affective network**   - Normalizing one’s experience through comparison with others - Taking ownership of one’s role and responsibilities - Experiencing emotions (positive or negative) conducive to learning | **Motivation**  **Ability**  **Opportunity** |
|  |  | **Recognition networks**   - Validating one’s knowledge and practice - Acquiring knowledge - Comparing one’s practice and knowledge - Consolidating knowledge |  |
|  |  | **Strategic networks**   - Developing templates for structuring data collection and clinical documentation - Identifying mental health assessment opportunities - Identifying solutions to improve nursing practice or care organization |  |
| **Week 6a**   - Answering reflective questions on integrating evidence-based mental health assessment practices into one's setting | - Support self-reflection | **Affective network**   - Developing self-awareness through reflection on one’s practice - Prepare for group activities | **Motivation** |
| **Week 6b**   - Discussing challenges related to adopting evidence-based mental health assessment practices | - Planning for organizational change and practice improvement with peers and coordinators/supervisors - Sharing knowledge and experiences with peers and experts | **Strategic networks**   - Identifying solutions to improve nursing practice or care organization | **Motivation**  **Opportunity** |
